# Evaluation of the Hemostatic Effect of an Innovative Tissue Adhesive during Extraction Therapy under Rivaroxaban in a Rodent Model

**DOI:** 10.3390/jfb14070333

**Published:** 2023-06-22

**Authors:** Marius Heitzer, Philipp Winnand, Anna Bock, Mark Ooms, Marie Sophie Katz, Kristian Kniha, Oliver Grottke, Frank Hölzle, Ali Modabber

**Affiliations:** 1 Department of Oral and Cranio-Maxillofacial Surgery, University Hospital RWTH Aachen, Pauwelstraße 30, 52074 Aachen, Germany; 2 Clinic for Anesthesiology/Operative Intensive Care Medicine, University Hospital RWTH Aachen, Pauwelstraße 30, 52074 Aachen, Germany

**Keywords:** tissue adhesives, tooth extraction, rivaroxaban, postoperative hemorrhage, anticoagulants

## Abstract

An increase in rivaroxaban therapies is associated with increased numbers of postoperative bleeding despite the use of hemostatic sponges, which are currently the gold standard treatment. VIVO has shown promising hemostatic results, favorable tissue properties, and ease of application, although it has not yet been used in the oral cavity. The aim of this study was to evaluate the hemostatic properties of VIVO in the extraction sockets of 31 rodents and compare this to gelatin sponge (GSP) therapy. At rivaroxaban concentrations of 264.10 ± 250.10 ng/mL, 62 extraction sockets were generated, of which 31 were treated with VIVO and 31 with GSP. The duration time, early and late bleeding events, and wound healing score were determined. Histologic examinations of the tissues were performed after 5 days. VIVO presented a longer procedure, 1.26 ± 0.06 min, but a significantly shorter bleeding time, 0.14 ± 0.03 min. There was no difference between the two groups in terms of the severity and timing of bleeding. More minor early bleeding events were observed for GSP. VIVO showed a significantly better healing score, with favorable histological results. In an animal study, VIVO showed promising hemostatic properties after tooth extraction under ongoing anticoagulative therapy.

## 1. Introduction

Postoperative bleeding is one of the most common post-interventional complications in ambulant dental treatment [1], accounting for up to 31% of cases [2]. Anticoagulants are among the most widely prescribed medications worldwide [3], and patients receiving systemic anticoagulation are at a particularly high risk of postoperative bleeding after dental extractions [1,4]. Conditions such as atrial fibrillation and thromboembolic disease often require anticoagulant therapy [5,6,7,8]. Despite the widespread use of these drugs, several drawbacks have been reported [8]. In the past few years, direct oral anticoagulants (DOACs) have been released on the market and are considered the drug of choice for long-term anticoagulation in the most common indications, such as thrombosis prophylaxis in atrial fibrillation, deep vein thrombosis, and pulmonary embolism, as well as their recurrence prophylaxis. The advantages over VKAs or low-molecular-weight heparins include ease of administration, improved efficacy, and less severe bleeding events [9]. In addition, DOACs result in less patient burden and lifestyle adjustment because they are independent of fluctuating dietary intakes of vitamin K and do not require regular routine monitoring of the coagulation parameters. In many Western countries, the use of DOACs has now surpassed that of VKAs [10].

Rivaroxaban belongs to the group of oxazolidinone derivatives that act competitively as direct inhibitors of free active factor X (F Xa), which is involved in the formation of thrombin in the coagulation cascade. Thus, this drug blocks the conversion of prothrombin into thrombin and prevents the formation of blood clots [11], which complicates clotting and, thus, the stopping of a bleeding wound after a tooth extraction due to this effect. Rivaroxaban is one of the various DOACs and currently accounts for the largest proportion (76%) of patients receiving DOAC medications during dental procedures [10]. In the literature, the risk of postoperative bleeding after tooth extraction under DOACs has been reported to be 26.9% [4]. In particular, rivaroxaban is described to have a significantly higher incidence of postoperative bleeding after dental extractions in medically compromised elderly patients (32.4%) when compared to other DOACs, such as apixaban, dabigatran, edoxaban, or even VAKs [12].

When patients require oral surgical treatment with oral anticoagulants (OATs), modifications to OAT medications are discussed [3,13]. It is possible to reduce the risk of postoperative bleeding by pausing drug therapy during the procedure. Controversial debates have taken place in the scientific literature [13,14,15], and different guidelines have been proposed as to whether anticoagulation should be interrupted. Nevertheless, it has been emphasized that the interruption of oral anticoagulation during these procedures could severely harm these patients in terms of an increased risk of embolic events [14,15]. Berton et al. described minimally invasive surgical therapies with ongoing anticoagulation therapy using local hemostatic measures and sutures applied to the extraction socket to reduce the risk of postoperative bleeding [13]. Among the hemostatic agents, oxidized cellulose, absorbable gelatin sponges, collagen sponges, fibrin glue, cyanoacrylate glue, platelet-rich plasma gel, calcium alginate, and topical thrombin have mainly been mentioned in the literature [16,17]. In particular, absorbable hemostatic sponges are a commonly used local hemostatic agent [16], and the use of these sponges has proven to be particularly effective.

In addition to the lack of approval for the clinical application of various hemostatic adhesives for oral surgery procedures [18], the use of fibrin glue after dental extractions in anticoagulated patients has been reported to have no advantage [19]. In addition to hemostasis, other requirements of adhesive preparations include the uncomplicated application and promotion of the wound healing of the extraction sockets [16]. Therefore, there is an ongoing search for simple and reliable hemostatic materials in the oral surgical procedures of patients with ongoing OATs.

The novel polyurethane-based tissue adhesive VIVO (Adhesys Medical GmbH, Aachen, Germany) is an innovative biodegradable adhesive that has demonstrated rapid and safe application [20]. It is an alternative to conventional hemostasis [21,22,23] with favorable tissue compatibility [24], as evidenced by good biocompatibility during the various degradation phases [23]. The authors hypothesized that the use of polyurethane-based VIVO leads to a decrease in postoperative bleeding under good wound healing conditions after dental removal under rivaroxaban therapy. This proof-of-concept study was conducted in a rat model under systemic anticoagulation using rivaroxaban in comparison to the established hemostatic measures using gelatin sponges (GSPs).

## 2. Materials and Methods

The study was performed according to German animal protection law and EU directive 2010/63. The animal protocol was approved by the Governmental Animal Care and Use Committee of the State of North Rhine–Westphalia (81-02.04.2020.A166). A total of 31 adult male Sprague-Dawley rats at 7 weeks of age weighing 250 g (Janvier Labs, Le Genest-Saint-Isle, France) were included in this study.

The animals were housed in a pathogen-free environment under a 12 h light/12 h dark cycle, with food and water ad libitum. According to an established protocol, the parenteral administration of rivaroxaban (3 mg/kg) at a therapeutic dose was performed 15 min before the surgical procedure [25]. Subsequently, the administration was repeated daily over a period of 5 days after surgery. To ensure equal drug levels between the animals, daily injections were given at the same time each day. Material and Machines can be found in Table S1

### 2.1. Blood Sample Determination

The calibration of the medication and verification of the rivaroxaban level was performed before the start of the experiment in three animals. For this purpose, after induction of anesthesia with isoflurane (5% by volume), and the continuation of inhalation anesthesia with isoflurane (1.5–2%) and oxygen as a carrier gas, 1 mL of blood was collected from the caudal vein via a nasal mask via a puncture using a 23 G needle. During the experiment, 1 mL of blood was obtained via the puncture of the caudal vein under general anesthesia before surgery and before finalization. Blood samples were collected in microsample tube sodium citrate (3.2%, Sarstedt). Prothrombin time (PT) (Hemosil Readiplastin), fibrinogen (thrombin reagent), and rivaroxaban concentrations (HemosIL Liquid Anti-Xa Assay using rivaroxaban calibrators and controls) were determined by standard laboratory methods using the appropriate tests (all from Werfen, Germany) on an ACL-TOP550 (Werfen, Germany).

### 2.2. Surgical Procedures

**Figure 1:**
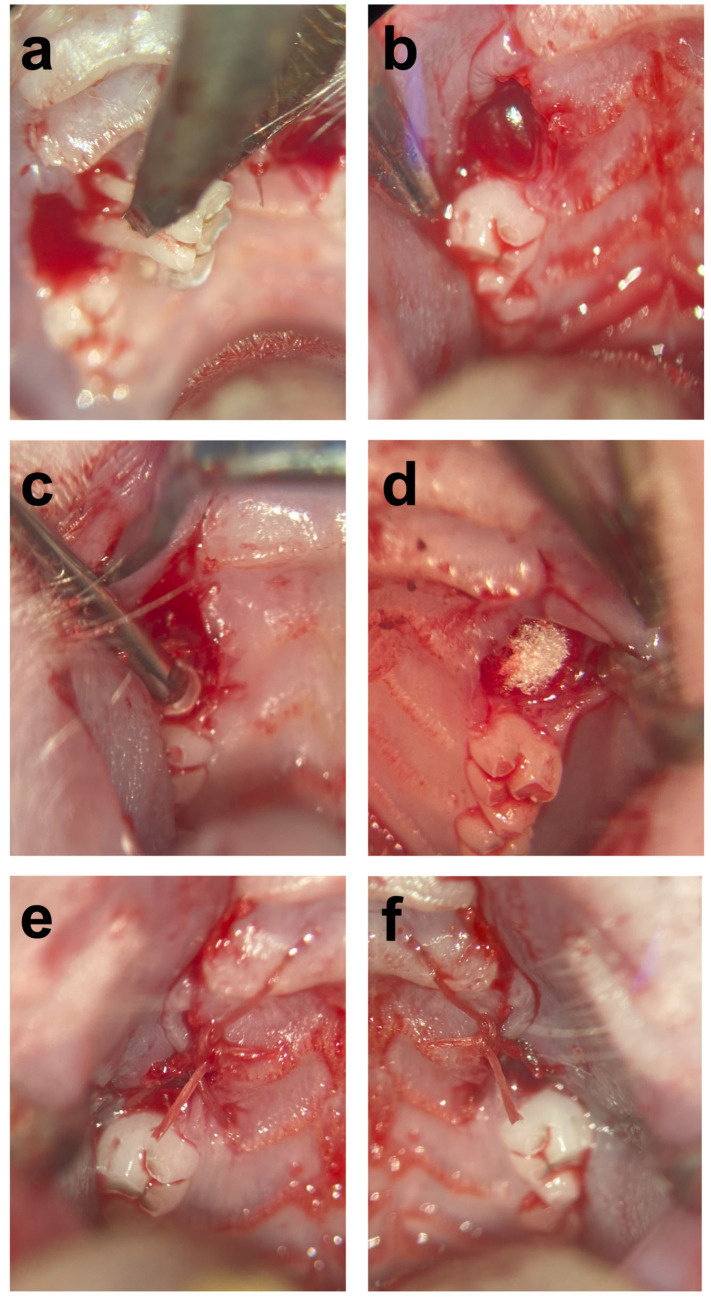
(**a**) Extraction of the first molar of the maxilla; (**b**) extraction socket; (**c**) application of VIVO, and (**d**) application of GSP inside of the extraction socket. (**e**,**f**) Adapted wound margins by means of the suture.

All surgical procedures were executed by the same person and carried out under an operation microscope (OPMI pico f170, Carl Zeiss AG, Oberkochen, Germany). The first molar was extracted under ongoing rivaroxaban medication in 31 rats using a split-mouth model. Under general anesthesia with medetomidine (0.25 mg/kg) and ketamine (80 mg/kg), the rats were placed in a supine position, and additional bilateral local anesthesia using a submucosal injection of Ultracaine 4% was initially performed before the extraction and osteotomy of the two first molars in the maxilla. The resulting extraction sockets on the right side were treated with VIVO adhesive. The left side was treated with a standard extraction socket restoration using GSP (ROEKO Gelatamp, Coltene, Altstätten, Switzerland). Subsequently, the wound margins of the extraction sockets were adapted with the development of a mucoperiosteal flap and standard sutures (Vicryl 6-0, Ethicon Inc., Raritan, NJ, USA; Figure 1). If there was persistent bleeding, slight pressure was applied with gauze until the bleeding stopped. One animal died after the operation under general anesthesia without bleeding. The time was measured for the two procedures after the tooth was removed, and the hemostatic treatment of the alveolus via GSP insertion or VIVO application was started. The operation time was stopped after the coverage of the extraction socket with a mucoperiosteal flap was completed. The time required for hemostasis was recorded immediately after finishing the operation and when the bleeding began to stop.

### 2.3. Clinical Examination

Oral bleeding events, including severity and timing, were recorded during thrice-daily oral cavity inspections. The degree of bleeding was categorized as minor, clinically relevant, or major according to an established protocol [4]. The categorization of the timing of bleeding was either early bleeding, which occurred immediately or the day after, or delayed bleeding, which occurred on Day 2 or afterward [25].

Wound healing was assessed according to the established Wound Evaluation Scale (WES; Table 1). The scale includes six clinical variables, each scored as 1/0 (absent/present), and the variables were subsequently added to the total score: (1) protruding wound edges; (2) contour irregularities (wrinkling); (3) distance between wound edges > 2 mm; (4) edge inversion (sinking, curling); (5) inflammation (redness, discharge), and (6) overall cosmetic appearance (good/not good). A score of 6 is considered the optimal wound situation of the extraction socket [26,27].

### 2.4. Histomorphometric Analysis

After the resection of the affected part of the maxilla, the samples were stored in 4% formalin (neutrally buffered with methanol; Otto Fischar GmbH & Co. KG, Saarbrücken, Germany) for 2 days, and then decalcification was carried out for 4 weeks at 37 °C by storing the samples in 20-fold volume ethylenediaminetetraacetic acid (EDTA; MolDecalcifer, Menarini, Florence, Italy). The EDTA solution was changed every 2 days. The paraffin-embedded resection parts of the maxilla were sectioned every 5 µm (in thickness) and stained with hematoxylin and eosin (H&E) according to the standard protocol. The tissue and extraction sockets were analyzed using ImageJ software (free Java software provided by the National Institute of Health, Bethesda, MD, USA). All examinations were blinded to the source groups, and histological analyses were performed according to established protocols.

### 2.5. Statistical Analysis

The sample size was calculated by using G*Power software (G*Power, Version 3.1.9, Düsseldorf, Germany, Faul et al. [28,29]). The a priori test (Wilcoxon–Mann–Whitney test for two groups) was used as an indication. Using a 0.05 significance level, an effect size of 0.66, and 80% power, at least n = 31 extraction sockets per group would be needed to verify the hypotheses.

All data were analyzed using GraphPad Prism 7.0 (GraphPad Software, Inc., La Jolla, San Diego, CA, USA) and were checked for normality distribution. For parametric statistics, the data that met the criteria of the D’Agostino–Pearson test and the Shapiro–Wilk test for normal distribution were used. The corresponding results were analyzed using an unpaired *t*-test. The Mann–Whitney U test was used for nonparametric independent variables to compare the differences between the parameters. All data represented the means ± standard deviation (SD), and statistical significance was assessed at a level of *p* ≤ 0.05.

## 3. Results

### 3.1. Blood Sample Determination

**Figure 2:**
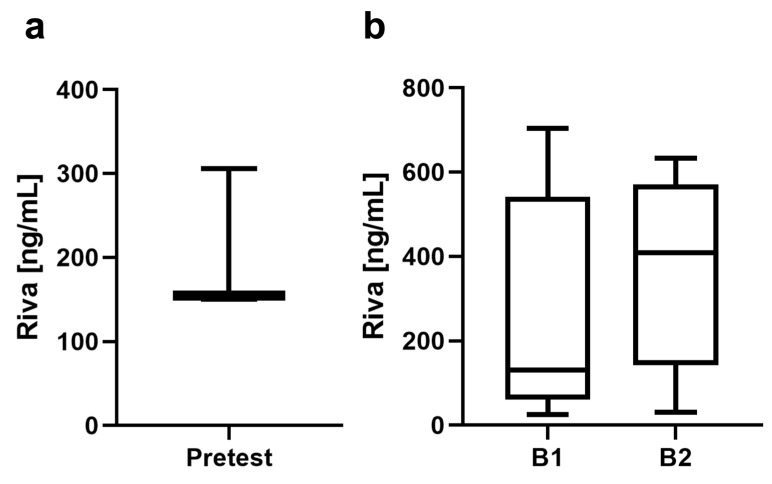
(**a**) Rivaroxaban concentration before the start of the experiment. (**b**) Rivaroxaban concentrations at blood drawing B1 before operation and B2 before the end of the experiment.

**Figure 3:**
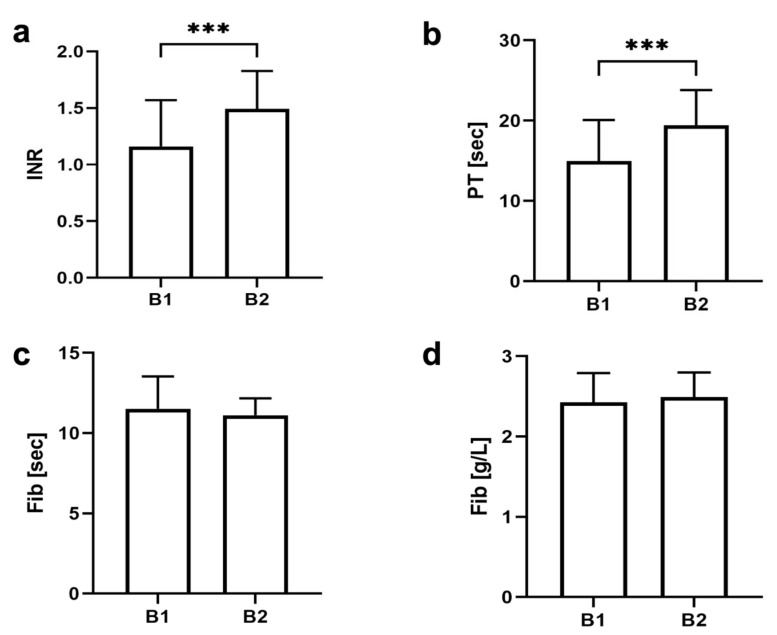
Graphical representation of the blood analysis. (**a**) Internationalized normal ratio (INR); (**b**) prothrombin time (PT); (**c**,**d**) measurement of fibrinogen; *** *p* ≤ 0.001.

In this study, a total of 62 maxillary first molars were extracted under ongoing anticoagulation therapy by rivaroxaban application and were evaluated in a split-mouth model. The preliminary studies to calibrate the rivaroxaban level showed a rivaroxaban concentration of 203.53 ± 88.86 ng/mL in three animals. Medication with rivaroxaban was then maintained for the main study, showing a rivaroxaban concentration at B1 of 264.10 ± 250.10 ng/mL before the intervention and a concentration at B2 of 356.00 ± 212.60 ng/mL in the venous blood of the rats after 5 days (Figure 2). The PT at B1 was 14.97 ± 5.11 and significantly prolonged at B2 at 19.44 ± 4.38 (*p* < 0.001). The measured INR at B1 was 1.16 ± 0.41, and at the second blood draw, it was 1.49 ± 0.33. No differences were recorded in fibrinogen concentrations in either blood drawing (Figure 3).

### 3.2. Surgical Procedures

**Figure 4:**
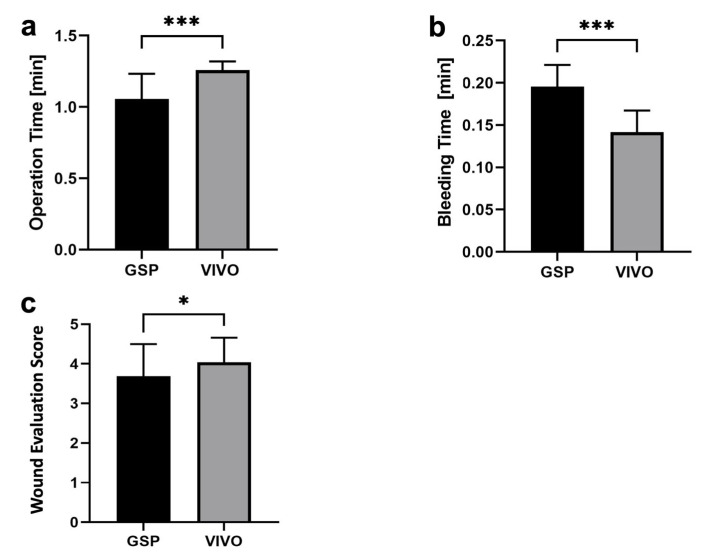
(**a**) Graphical representation of operation time, (**b**) bleeding time, and (**c**) Wound Evaluation Score; * *p* = 0.038, *** *p* ≤ 0.001.

Of the molar extraction sockets, 31 were treated by GSP, and 31 were treated by polyurethane-based adhesive for reasons of hemostatic treatment. Figure 4 shows that the operation time for the GSP group, with 1.06 ± 0.18 min, was significantly faster (*p* < 0.001) than the operation time for the polyurethane group, with 1.26 ± 0.06 min. The bleeding time was 0.14 ± 0.03 min, which was significantly shorter (*p* < 0.001) in the VIVO group when compared to the GSP group, with a bleeding time of 0.20 ± 0.03 min, respectively.

### 3.3. Clinical Examination

The clinical examination showed no significant difference between the two groups in terms of the degree of bleeding (*p* = 0.56) and the time point of bleeding (*p* = 0.56). In the GSP group, with 0.07 ± 0.26, two minor bleeding events occurred postoperatively. Both bleeding events occurred as early bleeding (Table 2 and Table 3). In the extraction alveolus treated with VIVO, overall, one minor bleeding and one early bleeding condition were observed with 0.03 ± 0.19. Late bleeding and clinically relevant major bleeding did not occur in either treatment group.

After a period of 5 days, the wound healing of the extraction sockets of the GSP and the VIVO groups was assessed by means of the WES healing score. Wound adaptation in the VIVO group clinically showed good adaptation of the wound margins, less redness of the soft tissue, and a good overall cosmetic appearance. This is shown by the evaluation of the WES score, with a significantly better healing score of 4.03 ± 0.63 (*p* = 0.38) compared to the GSP group, with an overall healing score of 3.69 ± 0.81 (Figure 4).

### 3.4. Histomorphometric Analysis

**Figure 5:**
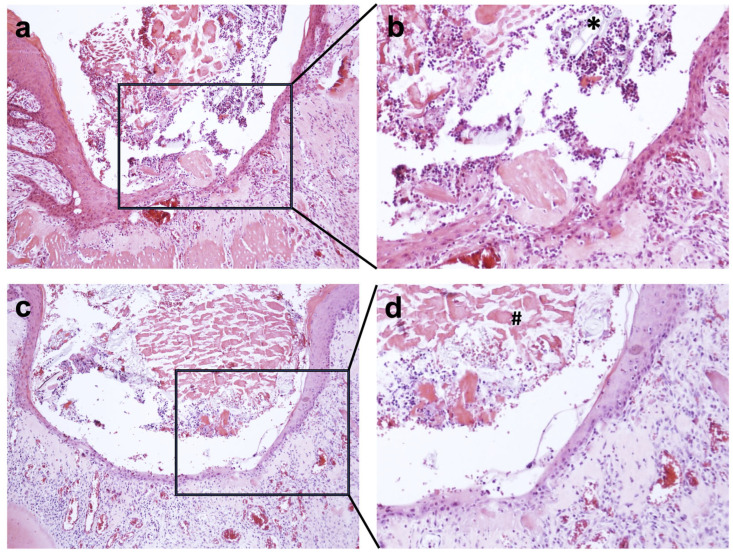
Histological images of extraction sockets of GSP * (**a**,**b**). (**c**,**d**) Shows histological slices of VIVO #. Magnification of (**a**,**c**) (10×). Magnification of (**c**,**d**) (20×).

In the histological sectional images of the GSP and VIVO groups, a stable thrombus with isolated accumulations of erythrocytes was present within the extraction sockets after 5 days (Figure 5). Inflammatory infiltrations were evident in the basal portions of the extraction sockets in both groups, although inflammatory infiltrations tended to be more prevalent in the GSP group. In addition, reticular remnants of the gelatin sponge were visible in the GSP group within the extraction socket after 5 days, whereas in the VIVO group, the adhesive was not clearly visible.

## 4. Discussion

The present study was the first to investigate the hemostatic effect of the polyurethane-based adhesive VIVO after dental extraction under rivaroxaban therapy. Furthermore, over a period of 5 days, the hemostatic treatment of the biodegradable adhesive was evaluated and compared to the gold standard, GSP, which served as a control. Many studies have indicated that the risk of bleeding after dental extractions should not be reduced by the administration of OAT drugs [3,18,30]; however, the paradigm has existed for more than 10 years that the risk of postoperative bleeding should be reduced by local hemostatic measurements [18]. Although local measurements were taken, there was still a described risk of bleeding after the dental extractions under an anticoagulative medication of 3.63–27% [31,32]. Therefore, it is essential to search for new therapeutic strategies to minimize the bleeding incidence of anticoagulated patients undergoing dental extractions.

Rodents are an established animal model for studying dental subjects [33,34] or surgical measurements under rivaroxaban therapy [35,36,37]. Different applications of rivaroxaban and varying concentrations have been described in the literature [35,36,37,38]. On the one hand, oral administration by gavage or feeding represents a realistic model for oral anticoagulative medication. On the other hand, Weinz et al. [38] and Parry et al. [35] illustrated a safe and reliable anticoagulative therapy via the intravenous application of rivaroxaban at a dosage of 3 mg/kg of body weight, which is not subject to the fluctuations of per os administration of animal food uptake, nor is it burdened by the life-threatening risk of intratracheal misapplication by gavage to the animals. Although the intravenous administration of an OAT presents different pharmacodynamics, the intravenous administration of rivaroxaban offers the advantage (over oral gavage) of not violating the sensitive wound conditions of the extraction alveoli, making post-extraction alveolar hemorrhage studies feasible over a 5-day period.

Guillou et al. described therapeutic anticoagulation using 3 mg/kg rivaroxaban in a Wistar rat infarct model at blood concentrations of 387.7 ± 152.3 ng/mL rivaroxaban [39]. Similarly, the rivaroxaban concentrations in the preliminary studies resulted in a rivaroxaban concentration of 203.53 ± 88.86 ng/mL. Likewise, venous rivaroxaban levels (preoperatively) of 264.10 ± 250.10 ng/mL and pre-final of 356.00 ± 212.60 ng/mL showed blood concentrations similar to human therapeutic levels [39]. Consistent with the findings of Yoshikawa et al., who described mean PT values of 14.2–17.9 in a study of postoperative bleeding after tooth extractions in patients treated with rivaroxaban [40], PT values of 14.97 ± 5.11 for B1 and 19.44 ± 4.38 for B2, respectively, were obtained in this study.

In the literature, a 32.4% risk of rebleeding has been reported in elderly patients after tooth extraction under rivaroxaban medication [12]. The rationale of the present study was to evaluate whether the use of polyurethane-based VIVO in oral surgery can reduce the risk of rebleeding when compared to the gold standard treatment during ongoing rivaroxaban medication. Both of the two rebleeding events in the GSP group occurred in a total of 31 extraction sockets, with a rebleeding risk of 6.45%, and a single bleeding event in the VIVO group, with a total of 3.23%, meaning that post-extraction bleeding events are far from the high rebleeding values reported in the literature. In a clinical study with 52 patients on post-extraction bleeding without hemostatic measurements, Micolette et al. reported no difference in the number of early bleeding events between patients without OATs when compared to patients with OATs, 69% of whom were taking rivaroxaban. In contrast, a significantly higher number of seven delayed bleeding events occurred after tooth extractions in patients taking OATs [4]. In this study, no delayed bleeding events were observed in either of the hemostatic treatment groups during ongoing rivaroxaban therapy. In the rodent study, the data showed appropriate hemostatic therapies for both treatment groups for tooth extraction under ongoing rivaroxaban therapy. However, while the absolute number of post-extraction bleeding events over the 5-day period was lower in the VIVO group compared to the GSP group, the difference was not significant. On the one hand, this could be due to the short postoperative observation period of 5 days, and on the other hand, it could be caused by the small number of 31 cases per group.

The duration of tooth extraction is another significant risk factor for the occurrence of postoperative complications after oral surgery [41], making a shortened operative time particularly important when taking OATs. The operation time of the extraction sockets treated with GSP was significantly faster at 1.06 ± 0.18 min when compared to the use of VIVO, with an operation time of 1.26 ± 0.06 min. On the other hand, the bleeding time at the end of the operation was significantly shorter in the VIVO group, at 0.14 ± 0.03 min, which makes the total time of both procedures closer together. Nevertheless, the GSP procedure represents the faster treatment of an extraction socket under ongoing anticoagulation.

The use of wound healing scales provides surgeons with the opportunity to monitor the course of wound healing in a standardized manner [26,27]. Furthermore, the quality of the healing response after oral and maxillofacial surgery is influenced by different conditions of wound closure [26], which can be compared and quantified using a wound healing score. In this trial, the monitoring of extraction sockets was assessed by means of the established WES [26,27]. The different conditions of wound closure were based on the varying local measurements of the GSP and VIVO groups. VIVO had a significantly higher WES, with 4.03 ± 0.63 (*p* = 0.38), according to a good adaptation of the wound margins, less redness of the soft tissue, and a good overall cosmetic appearance when compared to the GSP group. An increased bleeding tendency for gingiva when taking rivaroxaban has been described in the literature [42,43].

When considering that the inflammation of gingiva can lead to uncomfortable bleeding in the oral cavity [44] regardless of extractions, the WES is an important tool in the context of ongoing anticoagulant therapy.

The insertion of sponges made of gelatin is considered the gold standard treatment. The structure of the sponge provides mechanical stability to the coagulum, and the hemostatic effect of collagen inserts comprises the activation of the intrinsic coagulation pathway and aggregation of platelets upon contact with collagen [45]. Histological cross-sectional images showed correspondingly stable thrombus formations in extraction sockets, which were treated with GSP 5 days after surgery. Nevertheless, a major disadvantage of collagen sponges is that they are made of animal native type I and type III collagens [46], and these, like any xenogeneic preparation, pose an increased risk of allergic, immunologic, and even anaphylactic reactions [47]. In rat studies, VIVO showed sufficient hemostasis under moist conditions when applied in a sealing fashion to vascular anastomoses [20,24]. In the histologic evaluation, VIVO showed comparable thrombus formations when compared to extraction sockets treated with GSP. In addition, the biodegradable polyurethane-based adhesive showed favorable immunologic properties in long-term studies [23,24]. VIVO comprises two components: a polyurethane prepolymer with reactive isocynate groups and an amino-based curing agent. They are both fully synthetic, unlike commonly used medical adhesives [48]. As a result, the risk of allergic and immunological reactions to animal hemostatic preparations is lower.

Two limitations of this animal study are the short observation time (5 days) and that there these were exclusively studies in rodents with rivaroxaban, and there were no control groups without anticoagulation. In accordance with the 3Rs principle of limiting the number of animals (reduction) and their suffering (refinement), the total number of animals used was significantly reduced by omitting a control group without anticoagulation. Similar to the lack of a control group without anticoagulation, another limitation of the present study is that the two forms of therapy were evaluated by means of GSP and VIVO and were not compared to extraction alveoli without hemostatic measures, which, in accordance with the 3R principle, also resulted in a reduction in the experimental animals. In future studies, in addition to increasing the number of cases, a drug-treated control group and a therapeutic control group without material insertion should be introduced into the extraction socket. In addition, for an improved assessment of the wound healing of GSP and VIVO, future trials should be conducted over a longer trial period with a longer postoperative observation time.

## 5. Conclusions

In conclusion, against the background of a small animal study, we demonstrated the following:

- The use of the polyurethane-based biodegradable tissue adhesive VIVO offers promising results in reducing postoperative bleeding risk in oral surgery;
- After 5 days, VIVO showed better wound healing regarding extraction sockets;
- Future studies with a higher number of extraction alveoli are essential for the further evaluation of the incidence of rebleeding with OATs and therapy with VIVO or GSP before transferring to human clinical use;
- Additionally, the degradation of the adhesive and the interaction of its degradation products on bone healing over the long term should be determined;
- Further research is needed to fully assess the efficacy and long-term safety of VIVO as a hemostatic agent in extraction sockets.

## Figures and Tables

**Table 1:** Wound Evaluation Scale.

	Absent/Present
Protruding wound edges	1/0
Contour irregularities (wrinkling)	1/0
Distance between wound edges > 2 mm	1/0
Edge inversion (sinking, curling)	1/0
Inflammation (redness, discharge)	1/0
Overall cosmetic appearance (good/not good)	1/0

**Table 2:** Degree of bleeding.

Group	Degree of Bleeding Mean ± SD					
	Minor		Relevant		Major	
GSP (*n* = 31)	2/31 (*p* = 0.56)	0.07 ± 0.26	0/31	0.00	0/31	0.00
VIVO (*n* = 31)	1/31 (*p* = 0.56)	0.03 ± 0.19	0/31	0.00	0/31	0.00

GSP = Gelatine Sponge; VIVO = Polyurethane Adhesive.

**Table 3:** Time point of bleeding.

Group	Early Bleeding Mean ± SD		Delayed Bleeding Mean ± SD	
GSP (*n* = 31)	2/31 (*p* = 0.56)	0.07 ± 0.26	0/31	0.00
VIVO (*n* = 31)	1/31 (*p* = 0.56)	0.03 ± 0.19	0/31	0.00

GSP = Gelatine Sponge; VIVO = Polyurethane Adhesive.

## Data Availability

The data presented in this study are available upon request from the corresponding authors.

## Supplementary Materials

The following supporting information can be downloaded at: https://www.mdpi.com/article/10.3390/jfb14070333/s1, Table S1: Material and Machines.
