# Supplementary material for: Evaluation of the Hemostatic Effect of an Innovative Tissue Adhesive during Extraction Therapy under Rivaroxaban in a Rodent Model
Source: J Funct Biomater. 2023 Jun 22;14(7):333. doi: 10.3390/jfb14070333 (PMC10381264; doi:10.3390/jfb14070333)
Supplement: Supplementary file 1 [file jfb-14-00333-s001.zip › jfb-2436163-supplementary.pdf]

Table S1. Material and Machines.

	Manufacturer	Country	LOT/Model Number
Test tubes sodium citrate (3.2%)	Sarstedt	Germany	41.1506.002
Hemosil Readiplastin	Werfen	Germany	00020301400
Thrombin reagent	Werfen	Germany	0009758515
HemosIL Liquid Anti-Xa Assay	Werfen	Germany	0020302601
ACL-TOP550	Werfen	Germany	00000280045
OPMI pico f170	Carl Zeiss	Germany	01SYSD9
ROEKO Gelatamp	Coltene	Switzerland	60022234
Vicryl 6-0	Ethicon Inc.	USA	1705202
VIVO adhesive	Adhesys Medical GmbH	Germany	N.A.
4% Formalin	Otto Fischar GmbH & Co. KG	Germany	27279
Hematoxylin and eosin	Carl Roth	Germany	9194.2

N.A. not applicable
